# The Effect of Conditioned Media of Stem Cells Derived from Lipoma and Adipose Tissue on Macrophages’ Response and Wound Healing in Indirect Co-culture System In Vitro

**DOI:** 10.3390/ijms20071671

**Published:** 2019-04-03

**Authors:** Sanja Stojanović, Stevo Najman

**Affiliations:** Department of Biology and Human Genetics and Department for Cell and Tissue Engineering, Faculty of Medicine, University of Niš, 18000 Niš, Serbia; stevo.najman@medfak.ni.ac.rs

**Keywords:** lipomas, adipose tissue, stem cells, macrophages, immunomodulation, wound healing, in vitro

## Abstract

Immunomodulatory and wound healing activities of adipose-derived stem cells (ADSCs) have been reported in various in vitro and in vivo experimental models suggesting their beneficial role in regenerative medicine and treatments of inflammatory-related disorders. Lipoma-derived stem cells (LDSCs) were reported as a potential tool in regenerative medicine due to the similarity with ADSCs but we have previously shown that LDSCs have different differentiation capacity than ADSCs despite a similar mesenchymal phenotype. To further analyze the potential differences and/or similarities between those two stem cell types, in the present study we examined the macrophages (MΦs)’ response, immunomodulatory and wound healing effect of conditioned media (CM) of LDSCs and ADSCs in indirect co-culture system in vitro. We confirmed similar mesenchymal phenotype and stemness state of LDSCs and ADSCs but indicated differences in expression of some inflammatory-related genes. Anti-inflammatory potential of CM of LDSCs and ADSCs, with pronounced effect of LDSCs, in unstimulated RAW 264.7 MΦs was evaluated by decrease in *Tnf* and increase in *Il10* gene expression, which was confirmed by corresponding cytokines’ secretion analysis. Conditioned media of both LDSCs and ADSCs led to the functional activation of MΦs, with slightly more pronounced effect of CM of LDSCs, while both stimulated wound healing in vitro in a similar manner. Results of this study suggest that LDSCs secrete soluble factors like ADSCs and therefore may have a potential for application in regenerative medicine, due to immunomodulatory and wound healing activity, and indicate that LDSCs through secretome may interact with other cells in lipoma tissue.

## 1. Introduction

Adipose-derived stem cells (ADSCs) represent a great tool for application in tissue engineering and regenerative medicine due to their self-renewal potential, proliferation capacity and potential to differentiate into numerous cell types [1,2]. The great advantage of ADSCs compared to the other mesenchymal stem cells (MSCs) is that they can be isolated in large quantities from very abundant and easily accessible adipose tissue [3]. In addition to their ability to provide different cell types and possess regenerative potential [4,5,6,7], immunomodulatory activity of ADSCs, and MSCs in general, in vitro and in vivo has been reported that considerably enlarges the application field of these cells for the treatment of inflammatory diseases and autoimmune disorders [8,9,10,11]. MSCs can express immunomodulatory activity through direct contact with immune cells or through secretion products [12]. MSCs can exert both anti-inflammatory and pro-inflammatory properties, which is dependent on the factors from microenvironments that “license” the MSCs. The MSC secretome and following activity varies depending on the cell activation state, the cytokines and other molecules to which cells are exposed [13]. It has also been shown that pro-inflammatory stimuli upregulate the secretion of anti-inflammatory factors by MSCs which is one of the mechanisms of their action [14]. When tissue damage occurs, it is followed by production of pro-inflammatory factors that induce migration of different cells including MSCs to the damaged site. MSCs then respond to these stimuli by producing anti-inflammatory cytokines and different growth factors that help damaged tissue to heal [15,16]. By down-regulating the inflammatory response MSCs favor the tissue repair processes [17]. Immunosuppressive activity of MSCs has mostly been reported which together with low immunogenicity makes them good candidates for transplantation with no need for matching donor and recipient [14]. MSCs can also be induced to produce pro-inflammatory stimuli and to promote immune response not only to induce immunosuppression, which is mainly orchestrated and dependent on the microenvironment and inflammation status of the tissue [16]. It has been reported that ADSCs secrete various soluble factors such as cytokines, chemokines and growth factors, constitutively and when are stimulated with different stimuli, both in vitro and in vivo [10,12,15,16,18]. Besides these molecules, MSCs secrete exosomes and microvesicles that contain different biologically active molecules such as miRNAs, lipid molecules and various proteins [18,19]. The published data regarding secretion products of MSCs indicates the possibility for development of cell-free therapeutic strategies based on the MSC secretome as an alternative to cell-therapy approaches [18,19,20]. Wound healing effect of ADSCs has also been reported both in vitro and in vivo, based on direct acting of cells through differentiation into fibroblasts, keratinocytes, osteoblasts etc., or through secretory products presented in conditioned media [21].

Lipomas are benign adipose tissue tumors that represent one of the most common soft tissue neoplasms of mesenchymal origin, with insufficiently clarified etiology and pathogenesis. Several studies reported that lipoma tissue is a good source of stem cells [22,23,24]. Lipoma-derived stem cells (LDSCs) have been proposed by some authors as a potential tool in regenerative medicine due to similarities with ADSCs in term of proliferation capacity and differentiation potential towards osteoblasts, adipocytes and chondrocytes [22,23,24]. The use of LDSCs could be very attractive since it would represent so called “recycling the useless tissue” and its application in regenerative medicine. It is also considered that stem cells may be responsible for the formation of bone and cartilaginous structures within lipoma implying their role in lipoma pathogenesis [25]. Recently, we have shown for the first time that LDSCs have a similar mesenchymal phenotype as ADSCs but different differentiation capacity towards adipocytes and osteoblasts due to the distinct molecular signature [26]. This could significantly limit the use of these cells for regenerative purposes but, on the other hand, detailed characterization of LDSCs moves us closer to revealing the mechanisms of lipoma formation.

Our aim to analyze inflammatory-related gene expression profile and effects of secretory products of LDSCs on macrophages’ response, as well as immunomodulatory and wound healing potential of LDSCs in indirect co-culture system in vitro, in comparison with ADSCs, arose from the following facts: (1) there is a need to further examine the characteristics of these cells in order to provide the possible mechanisms of their involvement in pathogenesis of lipomas; (2) the inflammation could be one of the triggers for tumor formation and, on the other side, guides tissue regeneration and reparation; (3) stem cells are more than involved in responding to inflammation environment and orchestrating further events in the tissue, and (4) potential application of LDSCs in biomedical field is questionable and still being sought.

According to our knowledge, apart from the data on proliferation and differentiation potential, there are no studies on immunomodulatory and wound healing activities of LDSCs, so these are the first in vitro data, which could indicate new areas of their potential use in regenerative medicine and give us closer insight into the molecular mechanisms of lipoma formation.

## 2. Results

### 2.1. Mesenchymal Stem Cell Phenotype and Expression of Stemness-Related Markers

**Figure 1:**
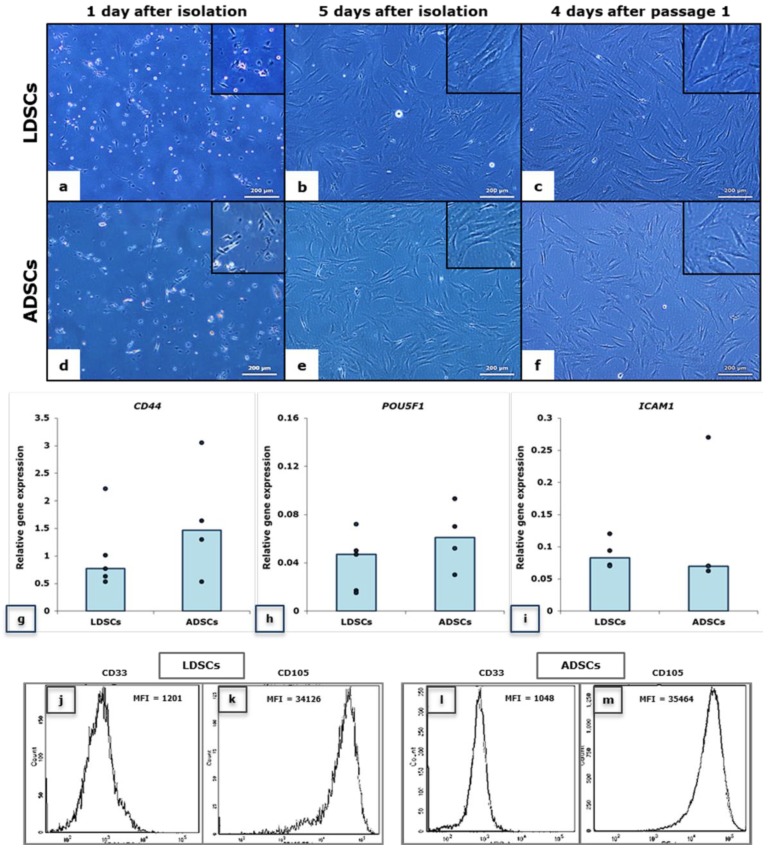
Morphology of lipoma-derived stem cells (LDSCs) (**a**–**c**) and adipose-derived stem cells (ADSCs) (**d**–**f**); images were acquired at day 1 (**a**,**d**), at day 5 after isolation (**b**,**e**) and at day 4 after passage 1 (**c**,**f**), on phase contrast with objective magnification 10×, cells are spindle-like in shape which is typical for mesenchymal stem cells (**b**,**c**,**e**,**f**); Relative expression of *CD44* (**g**), *POU5F1* (**h**) and *ICAM1* (**i**) genes in LDSCs and ADSCs at passage 2, normalized to *GAPDH*, presented as scatterplots with median, each dot represents one patient-derived cell culture per group, sample size for *CD44* and *POU5F1*: n(LDSCs) = 5 and n(ADSCs) = 4, for *ICAM1*: n(LDSCs) = 4 and n(ADSCs) = 4; Flow cytometric analysis of CD105 (**k**,**m**) and CD33 (**j**,**l**) cell surface marker expression in LDSCs (**j**,**k**) and ADSCs (**l**,**m**) at passage 2 (representative histograms per each group of samples), MFI—mean fluorescence intensity.

In Figure 1 the morphology of LDSCs (a–c) and ADSCs (d–f) is presented at three time points, 1 and 5 days after isolation and 4 days after passage 1. No significant differences were observed in morphology between LDSCs and ADSCs at those time points. Real-time polymerase chain reaction (PCR) analysis of *CD44* and *POU5F1* stem cell markers’ expression (Figure 1g,h) confirmed that both LDSCs and ADSCs express these genes at passage 2. Slightly higher expression of *CD44* and *POU5F1* in ADSCs compared to LDSCs was noticed, but was not statistically significant. Flow cytometric analysis (Figure 1j–m) revealed high expression of CD105, positive surface stem cell marker, in both LDSCs (Figure 1k) and ADSCs (Figure 1m) at passage 2, and poor expression of CD33, negative stem cell marker (Figure 1j,l). Both LDSCs and ADSCs express *ICAM1* however, slightly higher expression, but not significantly higher, was noticed in LDSCs (Figure 1i).

### 2.2. Expression of Inflammatory-Related Genes in Stem Cells

**Figure 2:**
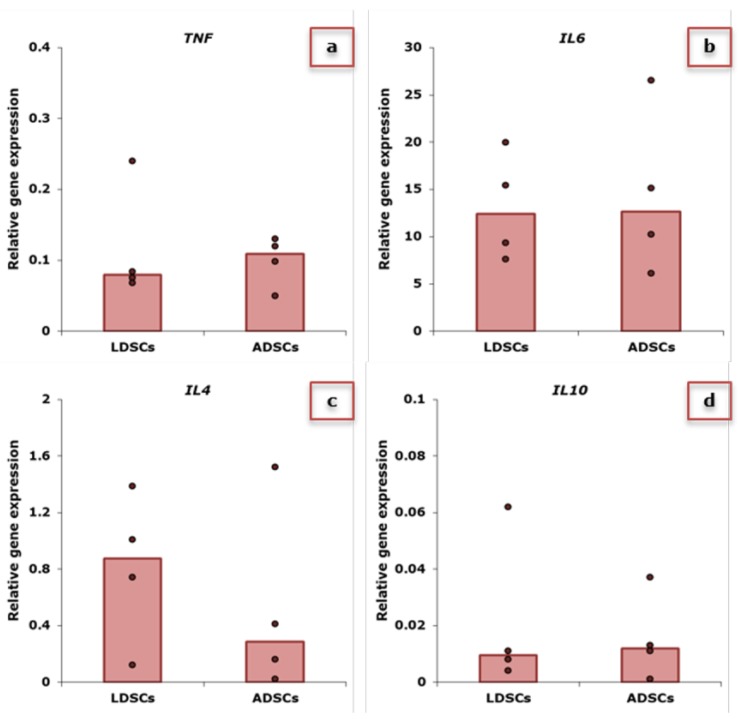
Relative expression of *TNF* (**a**), *IL6* (**b**), *IL4* (**c**) and *IL10* (**d**) genes in LDSCs and ADSCs at passage 2, normalized to *GAPDH*; scatterplots with median; dots represent patients’-derived cultures per group, sample size: n(LDSCs) = 4 and n(ADSCs) = 4 for all genes.

In Figure 2, relative expression of genes for pro- and anti-inflammatory cytokines in LDSCs and ADSCs at passage 2 is presented. Expression of *TNF*, *IL6*, *IL4* and *IL10* in LDSCs and ADSCs is similar with slightly higher *IL4* (Figure 2c) and lower *TNF* expression (Figure 2a) in LDSCs compared to ADSCs, although not statistically significant.

### 2.3. Macrophages’ Response to Conditioned Media of Lipoma-Derived Stem Cells (LDSCs) and Adipose-Derived Stem Cells (ADSCs)

**Figure 3:**
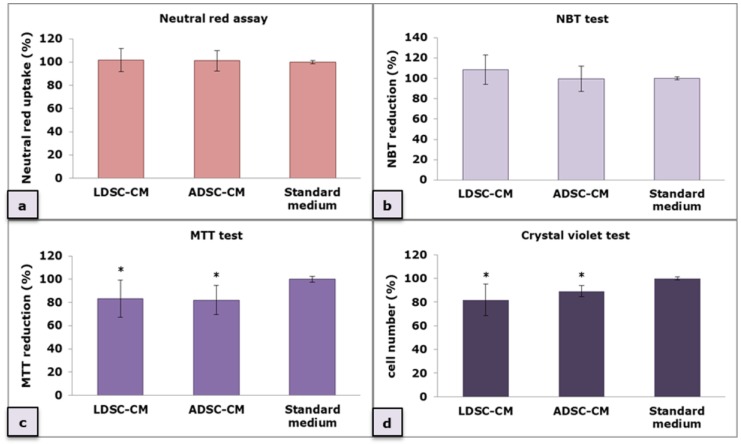
Macrophages’ response to LDSC-conditioned media (CM) and ADSC-CM evaluated by neutral red (NR) assay (**a**), NBT test (**b**), MTT test (**c**) and crystal violet (CV) test (**d**); mean ± standard deviation (SD); n(LDSCs) = 5 and n(ADSCs) = 4 (n − number of patients per group); for each patient sample culture-derived CM, as well as control culture, four to eight replicates were analyzed in each assay; (*) *p* < 0.05 (compared to standard medium).

Macrophages (MΦs)’ response to conditioned media (CM) of LDSCs (LDSC-CM) and ADSCs (ADSC-CM) after 48 h cultivation was evaluated through the RAW 264.7 MΦs’ ability to reduce NBT (detection of intracellular superoxide anion production in phagocytic cells) (Figure 3b) and MTT (the result of mitochondrial dehydrogenase activity) (Figure 3c) as well as to uptake neutral red (NR) dye (indicator of pinocytic activity) (Figure 3a). The crystal violet (CV) test (Figure 3d) was performed for cell number evaluation since the amount of bound dye directly correlates with the cell number. No statistically significant changes were observed between MΦs cultured in LDSC-CM and ADSC-CM in NR (*p* = 0.9) and NBT test (*p* = 0.29), however, when ratio between NR assay and CV test was calculated, as well as between NBT test and CV test (NBT reduction and NR uptake normalized to the cell number obtained by CV test for each sample) (Table 1), greater NR uptake (NR/CV (LDSC-CM) = 1.25 vs. NR/CV (ADSC-CM) = 1.13) and NBT reduction (NBT/CV (LDSC-CM) = 1.33 vs. NBT/CV (ADSC-CM) = 1.12) were observed in MΦs cultured in LDSC-CM than ADSC-CM, suggesting stronger functional activation of macrophages in the presence of LDSCs secretion products than ADSCs. Slightly lower reduction of MTT was observed in both LDSC-CM and ADSC-CM but the ratio between MTT and CV did not indicate any changes (Table 1).

### 2.4. Immunomodulatory Activity of Conditioned Media of LDSCs and ADSCs

**Figure 4:**
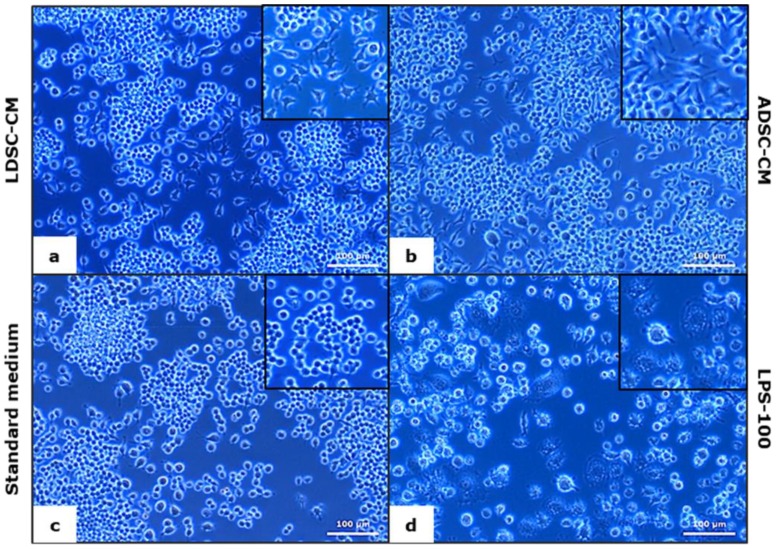
Morphology of RAW 264.7 macrophages cultured for 48 h in LDSC-CM (**a**), ADSC-CM (**b**), standard medium (**c**) and LPS-100 (**d**); phase contrast with objective magnification 20×.

After 48 h of RAW 264.7 MΦs cultivation in LDSC-CM and ADSC-CM, changes in cell morphology were noticed (Figure 4). Unlike control culture (standard medium) where cells were predominantly small and round in shape (Figure 4c), LDSC-CM (Figure 4a) and ADSC-CM (Figure 4b) induced phenotypic changes toward larger, spread shapes with extensions. LPS-100 activated cells (Figure 4d) were epithelial in shape and full of vesicles.

**Figure 5:**
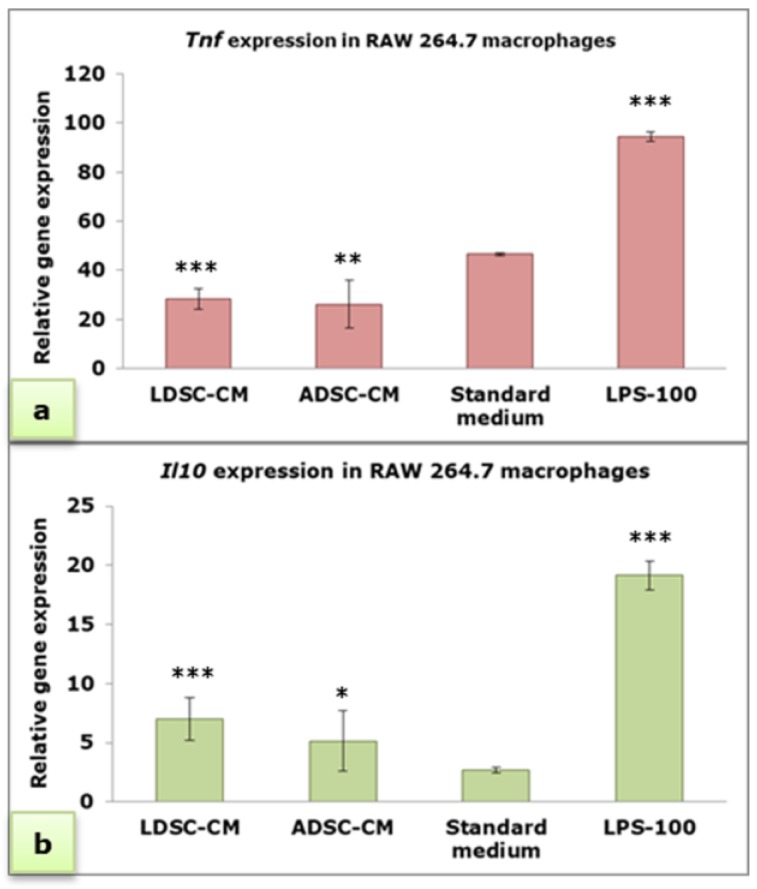
Relative expression of *Tnf* (**a**) and *Il10* (**b**) genes in RAW 264.7 macrophages cultured for 48 h in LDSC-CM, ADSC-CM, standard medium and LPS-100, normalized to *Actb*; mean ± standard deviation (SD); n(LDSCs) = 4 and n(ADSCs) = 4 (n − number of patients per group); for each patient sample culture-derived CM, as well as controls, four replicates were analyzed; (*) *p* < 0.05, (**) *p* < 0.01, (***) *p* < 0.001 (compared to standard medium).

Real-time PCR analysis (Figure 5) showed that both LDSC-CM and ADSC-CM significantly decrease the expression of *Tnf* (pro-inflammatory cytokine gene) compared to the standard medium and LPS treatment (Figure 5a), with no significant difference between stem cell-derived CM. By contrast, both LDSC-CM and ADSC-CM significantly increase the expression of *Il10* (anti-inflammatory cytokine gene) compared to the standard medium (Figure 5b).

**Figure 6:**
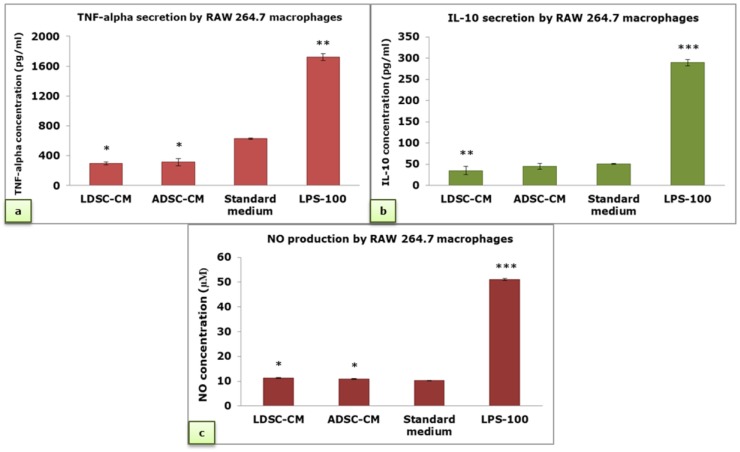
TNF-alpha (**a**), IL-10 (**b**) and NO concentration (**c**) in cell culture supernatant of RAW 264.7 macrophages cultured for 48 h in LDSC-CM, ADSC-CM, standard medium and LPS-100; mean ± standard deviation (SD); sample size: n(LDSCs) = 4 and n(ADSCs) = 4 (n − number of patients per group); for each patient sample culture-derived CM, as well as controls, four replicates were analyzed; (*) *p* < 0.05, (**) *p* < 0.01, (***) *p* < 0.001 (compared to standard medium).

Secretion of TNF-alpha by RAW 264.7 MΦs was significantly decreased in LDSC-CM and ADSC-CM compared to the standard medium and LPS-100, without a significant difference between types of CM (Figure 6a). There were no significant changes in IL-10 secretion between MΦs cultured in LDSC-CM and ADSC-CM, but compared to the standard medium IL-10 secretion was slightly lower in LDSC-CM (Figure 6b). Compared to LPS-100, concentration of IL-10 was significantly lower in all examined media. Slight, but significant, increase in nitric oxide (NO) production was noticed when MΦs were cultured in LDSC-CM and ADSC-CM compared to the standard medium (Figure 6c), but still significantly less than in LPS-100 treatment.

### 2.5. L929 Bioassay

**Figure 7:**
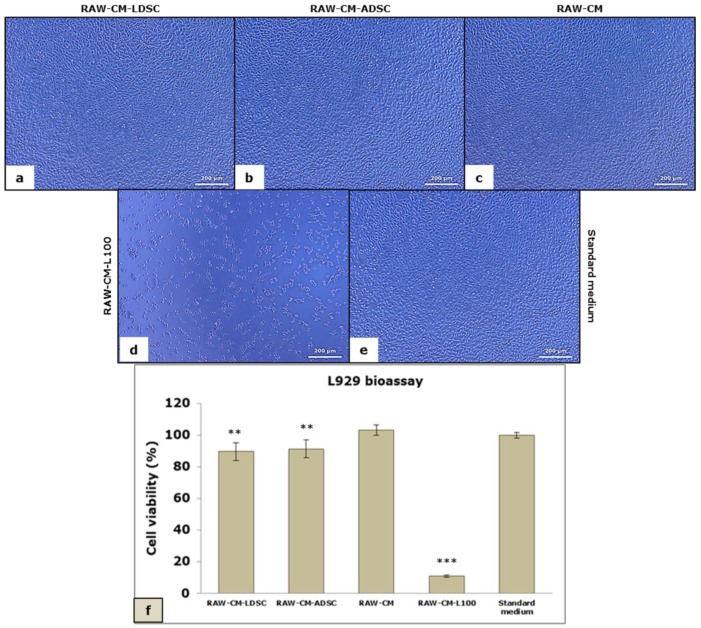
Morphology of L929 cells cultured for 24 h in conditioned media of RAW 264.7 macrophages previously cultured in LDSC-CM (RAW-CM-LDSC) (**a**), ADSC-CM (RAW-CM-ADSC) (**b**), standard medium for RAW cells (RAW-CM) (**c**), LPS-100 (RAW-CM-L100) (**d**) and standard medium for L929 cells (**e**); phase contrast with objective magnification 10x; viability of L929 cells in L929 bioassay evaluated by MTT test (**f**), mean ± standard deviation (SD); sample size: n(LDSCs) = 4 and n(ADSCs) = 4 (n − number of patients per group); for each patient sample culture-derived CM, as well as controls, four to eight replicates were analyzed; (**) *p* < 0.01, (***) *p* < 0.001 (compared to standard medium).

L929 bioassay, used to evaluate the pro-inflammatory cytokine-mediated cytotoxicity, revealed no changes in the number and morphology of L929 fibroblasts when cultured for 24 h in CM of RAW 264.7 MΦs that were previously cultured in LDSC-CM (RAW-CM-LDSC) (Figure 7a) and ADSC-CM (RAW-CM-ADSC) (Figure 7b) compared to CM of MΦs cultured in standard medium (RAW-CM) (Figure 7c) and standard medium for L929 cells (control culture) (Figure 7e), while significantly reduced number of cells, and predominantly apoptotic cells can be seen in the treatment with CM from MΦs stimulated with 100 ng/mL LPS (RAW-CM-L100) (Figure 7d) which was confirmed by the MTT test. MTT test (Figure 7f) revealed no significant changes between RAW-CM-LDSC and RAW-CM-ADSC, as well as RAW-CM and standard medium, but compared to the standard medium and RAW-CM all other media significantly reduced L929 cell viability.

### 2.6. In Vitro Wound Healing Analysis

**Figure 8:**
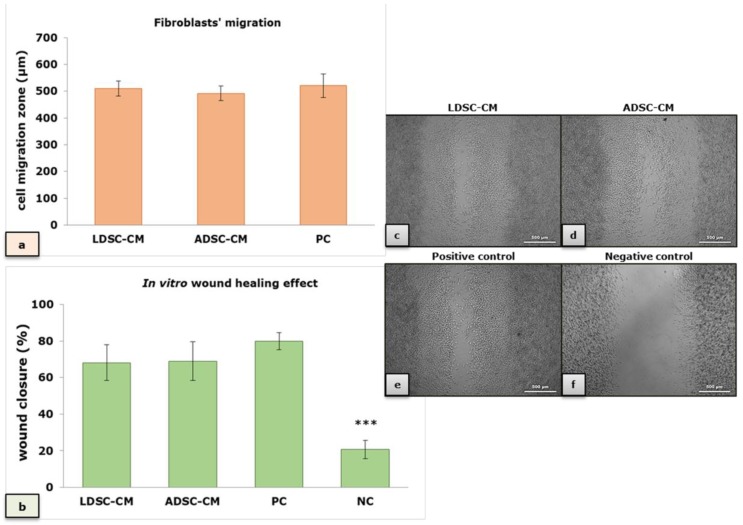
In vitro wound healing effect of conditioned media of LDSCs and ADSCs; effect of LDSC-CM, ADSC-CM, positive (PC) and negative (NC) control on fibroblasts’ migration (**a**) and wound closure (**b**); mean ± standard deviation (SD); sample size: n(LDSCs) = 4 and n(ADSCs) = 4 (n − number of patients per group); for each patient sample-derived CM, as well as controls, four replicates were analyzed; microscopic appearance of the “wound” 3 days after cultivation in LDSC-CM (**c**), ADSC-CM (**d**), positive control (**e**) and negative control (**f**); phase contrast with objective magnification 5×; (***) *p* < 0.001 compared to LDSC-CM, ADSC-CM and PC, no significant difference observed between both CM types and PC.

Fibroblasts’ migration (Figure 8a) and wound closure in vitro (Figure 8b) in the LDSC-CM and ADSC-CM were comparable and not significantly different than positive control (PC), but significantly stimulated compared to negative (NC) control (standard medium without serum), indicating stimulatory effect of CM of both LDSCs and ADSCs on wound healing. Microscopic appearance of the wound closure, after 3 days of cultivation in different media, is presented in Figure 8c–f.

## 3. Discussion

To be considered as stem cells, MSCs should meet some criteria that are proposed by Dominici et al. [27]. Both LDSCs and ADSCs in our study meet these criteria: they are adherent, had specific mesenchymal-like morphology when cultured in vitro, express specific MSC markers and as we have previously shown they can differentiate towards adipocytes and osteoblasts [26]. Although some authors reported that ADSCs had consistent morphology while LDSCs did not [28], several studies have shown that the morphology of LDSCs is very much like ADSCs and there were no morphological differences between those two cell types after long-term culture [22,23]. In our study the morphology of LDSCs and ADSCs was very similar and typical mesenchymal-like, without significant changes between them during cultivation (Figure 1a–f). According to the review provided by Mafi et al. [29], CD44 and CD105 are the most commonly reported positive cell surface markers of MSCs, which we also used in our study. Both LDSCs and ADSCs in our study highly express CD105 (evaluated by flow cytometry) (Figure 1k,m) and *CD44* (evaluated by gene expression analysis) (Figure 1g), while expression of CD33 was very low (Figure 1j,l) which is in accordance with other authors who reported CD33 as a negative MSCs marker [30,31]. The *POU5F1*, gene for Oct4, a pluripotent embryonic stem cell marker, was expressed in both LDSCs and ADSCs in our study (evaluated by gene expression analysis), with no statistically significant difference when compared (Figure 1h). Although there is lack of data in the literature on Oct4 expression in LDSCs, there are some reports on *POU5F1* expression in lipoma tissue, and it was shown that this gene is up-regulated in lipoma compared to the normal adipose tissue [32]. In the same study up-regulation of *CD44* in lipoma tissue was also reported.

ICAM-1 (intercellular adhesion molecule 1), also known as CD54, is a membrane glycoprotein, the member of immunoglobulin superfamily of cell adhesion molecules (CAMs), that plays an important role in the interaction between cells, cell adhesion, migration and immune response. ICAM-1 was shown to be highly expressed in ADSCs and represents one of the positive MSCs markers [30,33,34]. ICAM-1 is one of the factors responsible for immunomodulatory activity of MSCs, and was reported that its overexpression enhances the immunosuppressive effects of MSCs [35]. In the presence of pro-inflammatory cytokines, MSCs secrete high concentrations of various chemokines and express high levels of ICAM-1 that makes MSCs to become immunosuppressive [36]. ICAM-1 is reported to be a molecular switch responsible for activation of the immune suppressive activity of ADSCs [37]. There are no data on ICAM-1 expression in isolated LDSCs. Zavan et al. [32] reported that *ICAM1* was up-regulated in lipoma tissue compared to adipose tissue. In our study, *ICAM1* gene was slightly more expressed in LDSCs than in ADSCs, although not statistically significant, which could indicate potential immunosuppressive character of LDSCs. 

It is well known that MSCs secrete cytokines when stimulated by different factors from microenvironments which determine the response of MSCs towards pro-inflammatory or anti-inflammatory activity, but there are very contradictory reports in the literature about spontaneous secretion of cytokines from MSCs [10]. We analyzed gene expression profile of four cytokines, TNF-alpha, IL-4, IL-6 and IL-10, in both LDSCs and ADSCs at passage 2, just after collection of CM, to evaluate and compare gene expression levels in unstimulated cells and to determine basal inflammatory potential. We showed that all examined genes were similarly expressed with no statistically significant difference between LDSCs and ADSCs, probably due to variability among samples within the groups. Slightly higher expression of *IL4* (Figure 2c) and lower *TNF* expression (Figure 2a) was observed in LDSCs compared to ADSCs, suggesting potential anti-inflammatory character of LDSCs. When the cytokine secretion profile of human bone marrow (BM)-derived MSCs was analyzed on gene and protein expression level, it was found that IL-6 was highly expressed among 120 examined cytokines [38] which was also noticed in our study in both LDSCs and ADSCs (Figure 2b). Since MSCs are cells capable of activating and modulating immune response by secreting various cytokines and chemokines, it is expected that genes for those cytokines are already active to some extent which will enable quick response and secretion of cytokines to environmental stimuli. There are no data for isolated LDSCs but it has been shown that *IL6* and *TNF* are up-regulated in lipoma tissue compared to adipose tissue [32], which is probably due to the presence of various immune cells within the tissue. 

There are numerous reports on immunomodulatory, predominantly anti-inflammatory, activity of MSCs, and ADSCs, achieved through direct cell-to-cell interaction between MSCs and immune cells (lymphocytes, monocytes, macrophages etc.) or indirectly through secretion products of MSCs, in various models in vitro and in vivo. It was shown that murine ADSC-derived exosomes induce polarization of LPS and IFN-γ stimulated peritoneal macrophages’ (PMΦs) toward M2 MΦs as shown by increase in mRNA levels of M2 and decrease in M1 markers [39] while some studies reported that only pro-inflammatory cytokines induced-exosomes from ADSCs were able to significantly reverse the monocyte-derived MΦs’ phenotype from M1 towards M2, suggesting that immunomodulatory properties of ADSCs-derived exosomes are more likely to be induced by inflammatory microenvironments than to be constitutive [40]. Similar results were obtained when LPS and IFN-γ stimulated murine PMΦs were co-cultured with murine ADSCs or with ADSC-CM where CM evidently inhibited M1 polarization [41]. A decrease in TNFα and NO production in both stimulated and unstimulated MΦs, and an increase in IL-10 levels was noticed when murine PMΦs were incubated with apoptotic murine ADSCs in co-culture with and without LPS for 48 h [42]. When feline ADSCs were co-cultured with LPS-stimulated RAW 264.7 cells, pro-inflammatory cytokines TNF-α, IL-1β and iNOS were significantly decreased [43]. Similar findings were reported when CM of Oct4/Sox2 overexpressing ADSCs was examined on LPS stimulated RAW 264.7 cells [44]. Human ADSC-CM was reported to modulate the response of RAW 264.7 MΦs to LPS stimulation beneficially, by elevating the expression of IL-10 and decreasing the expression of pro-inflammatory cytokines [45]. Co-culturing of BM-MSCs with IFN-γ/LPS-stimulated BM-derived MΦs (BMDMs) significantly decreased the mRNA levels of M1 markers while enhanced the induction of IL-10 in IL-4-activated BMDMs, suggesting that MSCs switch MΦs from M1 to M2 phenotype [46]. Murine ADSC-CM and ADSC-CM supernatant decreased the expression of M1 markers in both LPS-stimulated and unstimulated BMDMs while it increased the expression of M2 markers in unstimulated BMDMs [47]. ADSC-CM exerted paracrine actions on differentiated human monocyte-derived MΦs to potentiate anti-inflammatory cytokines while it simultaneously reduced the pro-inflammatory cytokine TNFα [17].

We performed four different assays to assess the MΦs’ functional state after 48 h cultivation of unstimulated RAW 264.7 cells in LDSC-CM and ADSC-CM (Figure 3). We noticed that LDSC-CM enhances pinocytic activity of MΦs as evaluated by NR uptake, and NBT reduction was more pronounced in MΦs cultured in LDSC-CM than ADSC-CM, which could indicate that LDSCs produce soluble factors that activate and change the functional state of unstimulated RAW 264.7 MΦs to a greater extent than ADSCs. Changes in morphology of MΦs cultured in LDSC-CM and ADSC-CM were noticed compared to the standard medium, with MΦs become more spread and with extensions in CM of both stem cell types (Figure 4a,b) indicating changes in MΦs polarization towards reparative M2 phenotype. Gene expression analyses showed that *Tnf* expression decreased (Figure 5a) while *Il10* increased (Figure 5b) in MΦs cultured in LDSC-CM and ADSC-CM compared to standard medium and LPS treatment, which suggests that both LDSC-CM and ADSC-CM change the MΦs phenotype toward being anti-inflammatory. Measurement of cytokines’ secretion revealed that both LDSC-CM and ADSC-CM decreased the TNF-alpha secretion compared to standard medium and LPS treatment which is in accordance with the gene expression analyses and already discussed reports from other authors. No significant changes in IL-10 concentration were observed between CM and standard medium but increased mRNA levels suggest that maybe the cultivation period was not long enough for IL-10 to be secreted. All these results suggest that conditioned media of LDSCs and ADSCs switch RAW 264.7 MΦs towards anti-inflammatory M2 state, with more pronounced anti-inflammatory properties of LDSCs. NO production was slightly, but significantly, increased in both LDSC-CM and ADSC-CM compared to standard medium, with no difference between CM, and still significantly less than in LPS treatment. Since NO is produced in various physiological states, slightly higher concentrations in our study could not be addressed only to inflammatory response and probably represent the non-specific response to CM. In L929 bioassay, slight decrease in L929 cell viability in both RAW-CM-LDSC and RAW-CM-ADSC, but still significantly much less than RAW-CM-L100 (Figure 7f) could be due to slightly increased NO concentrations or the presence of other soluble products in MΦs-CM. Previously we reported that MΦs are the key actors in adipose tissue remodeling and dysfunction [48] which, together with the results obtained in this study, may imply that crosstalk between MΦs and stem cells could be one of the mechanisms involved in lipoma formation.

Numerous publications reported wound healing effects of ADSCs, and MSCs in general, on various models in vitro and in vivo that could be the result of direct-cell-cell interaction or paracrine effects through the secreted products [49]. MSCs secrete various factors such as growth factors, cytokines, and chemokines spontaneously or after stimulation, that are known mediators of tissue repair and key regulators of the wound healing process [50,51]. The stimulatory effect of ADSC-CM on proliferation, migration and wound healing in various in vitro models was reported, using keratinocytes and fibroblasts [52,53]. In vivo studies showed that ADSC, delivered in a biomimetic-collagen scaffold [54] or alone [55], enhances normal and diabetic wound healing. Conditioned media of rat BM-MSC was reported to enhance bone regeneration in rat calvarial model [56,57]. Antifibrotic effects of ADSCs, after fat grafting into the scar tissue, were reported to be achieved through different paracrine mechanisms and differentiation into fibroblasts and keratinocytes [58]. In our study, in an indirect co-culture wound healing model in vitro, enhanced wound closure (Figure 8b) and fibroblasts’ migration (Figure 8a) were observed with both LDSC-CM and ADSC-CM, comparable and not significantly different from positive control, suggesting stimulatory wound healing properties of CM of LDSCs and ADSCs, which supports other published data.

## 4. Materials and Methods

### 4.1. Tissue Sampling

Tissue samples used in this study were obtained at surgical clinics of the Clinical Center Niš, Serbia. Lipoma tissue samples were taken after surgical removal of solitary subcutaneous lipomas while subcutaneous adipose tissue samples were obtained from non-cancer patients during other surgeries. The study was approved by the Local Ethical Committee of the Faculty of Medicine, University of Niš, Serbia (approvals no. 01-6481-15, date 24.09.2013. and 12-6316-2/4, date 16.06.2016.) and all patients gave their informed written consent. Tissue samples from 10 patients were analyzed, among them 5 lipomas and 5 normal adipose tissue samples. Average age of patients with lipoma was 41.8 ± 7.1 while average age of non-lipoma patients was 47.2 ± 10.8. In the group of patients with lipoma, 3 were female and 2 were male, while in the non-lipoma group, 4 were female and 1 was male. Lipomas and adipose tissue samples were taken from several subcutaneous body depots: upper arm, back, neck, abdomen, hip and thigh. Body mass index (BMI) for all patients was less than 30, indicated non-obese patients.

### 4.2. Isolation and Cultivation of Mesenchymal Stem Cells

Both lipoma-derived stem cells (LDSCs) and adipose-derived stem cells (ADSCs) were isolated by enzymatic digestion of tissue samples, respectively, as we previously described [26]. Stromal vascular fraction (SVF) of cells, obtained from tissue homogenates after collagenase I digestion, was seeded in 25 cm^2^ cell culture flask (Greiner Bio One, Kremsmünster, Austria) in standard cell culture medium that contained Dulbecco’s modified Eagle’s medium (DMEM), 10% fetal bovine serum (FBS), 2 mM stable glutamine and 1% antibiotic-antimycotic solution (all purchased from Capricorn Scientific, Ebsdorfergrund, Germany). Media were changed 16–18 h after isolation to remove non-attached cells. After reaching confluency, the first cell passage was performed (P1), which enabled purification of mesenchymal stem cells. Cells were cultured in standard cell culture conditions, meaning temperature of 37 °C and humidified atmosphere with the presence of 5% CO_2_. Medium was changed every three days. Conditioned media (CM) of LDSCs (LDSC-CM) and ADSCs (ADSC-CM) were collected as a three-day medium just before passage 2 (P2) and stored at –80 °C until further analyses.

### 4.3. Cell Lines

For macrophages (MΦs)’ response and immunomodulatory analysis we used RAW 264.7 cell line which is commonly used cell line as an in vitro model for MΦs. To analyze the potential wound healing effect, and for L929 bioassay, we used L929 cell line which is commonly used as an in vitro model for fibroblasts. Both cell lines were purchased from the American Type Culture Collection (ATCC).

### 4.4. Light Microscopy

Cells in all assays were monitored on inverted light microscope (Observer Z1, Carl Zeiss, Oberkochen, Germany), under phase contrast. The images were acquired using the camera AxioCam HR in a software ZEN 2 blue edition (Carl Zeiss, Germany).

### 4.5. Flow Cytometry

Expression of positive (CD105) and negative (CD33) markers of mesenchymal stem cells was analyzed by flow cytometry, on both LDSCs and ADSCs, respectively, at P2. Cells were stained for 15-20 min with PE conjugated anti-CD105 human antibody (Clone 43A4E1, Miltenyi Biotec, Bergisch Gladbach, Germany) and APC conjugated anti-CD33 human antibody (Clone P67.6, BD Biosciences, Heidelberg, Germany) at 4 °C. After washing steps, cells were re-suspended in a buffer and analyzed on BD LSRFortessa™ cell analyzer with BD FACSDiva™ software v8 (BD Biosciences, Heidelberg, Germany).

### 4.6. Macrophages’ Response Assays

RAW 264.7 MΦs were seeded in 96 well plates in a density 0.5 × 10^4^ cells per well per 100 µL of standard medium (DMEM containing 10% FBS, 2 mM stable glutamine and antibiotic-antimycotic). After 24 h, 100 µL of LDSC-CM and ADSC-CM, respectively, was added to the cells (providing 50% final dilution of conditioned media of stem cells). Cells were cultured in LDSC-CM and ADSC-CM for 48 h in standard cell culture conditions which represented indirect co-culture system in vitro. As a control, RAW 264.7 MΦs were cultured in standard medium. When incubation period ended, MTT test, NBT test, Neutral red (NR) assay and Crystal violet (CV) test were performed. In MTT test, 100 µL of 1 mg/mL MTT (3-[4–dimethylthiazole-2-yl]-2,5-diphenyltetrazolium bromide, Carl Roth, Karlsruhe, Germany) solution per well was added to the cells and incubated for 2 h at 37 °C. Formed formazan crystals were dissolved by 100 µL of 2-propanol (Thermo Fisher Scientific, Waltham, MA, USA) and absorbance was measured at 540 nm on multichannel spectrophotometer (Multiskan Ascent plate reader, ThermoLab Systems, Helsinki, Finland). In NBT test, 100 µL of 1 mg/mL NBT (Nitro blue tetrazolium chloride, Sigma, St. Louis, MO, USA) solution per well was added to the cells and incubated for 1 h at 37 °C. Formed formazan deposits were dissolved by overnight incubation in 100 µL of 10% sodium dodecyl sulfate (SDS) in 0.01 M hydrochloric acid (HCl). Absorbance was measured at 550 nm. Neutral red assay was performed by incubation of cells in 100 µL of 0.1 mg/mL NR (neutral red, Sigma) solution per well, for 30 min at 37 °C. After that NR dye was dissolved in 1% acetic acid/50% ethanol solution and absorbance was measured at 540 nm. For the determination of cell number, cells were stained with 0.1% solution of CV dye for 10 min at room temperature (RT). Dye was dissolved with 33% acetic acid solution and spectrophotometrically quantified at 550 nm. In all assays (MTT, NBT, NR and CV) results were calculated and presented as percentages of the control (values for cells cultured in standard medium). The ratio between NR assay and CV test, NBT test and CV test as well as MTT test and CV test was calculated to normalize NR uptake, NBT and MTT reduction to the cell number obtained by CV test for each sample.

### 4.7. Immunomodulatory Assay

For immunomodulatory assay, RAW 264.7 MΦs were seeded in 24 well plates at density 2.5 × 10^4^ cells per well. After 24 h, equal volumes of LDSC-CM and ADSC-CM were added to the cells (providing 50% final dilution of CM of stem cells). Macrophages were cultured in LDSC-CM and ADSC-CM for the next 48 h in standard cell culture conditions, as indirect co-culture system. As a positive control for immunomodulation MΦs were cultured in 100 ng/mL of lipopolysaccharide (LPS) (LPS from *Escherichia coli* O111:B4, Sigma-Aldrich) while the negative control were cells cultured in standard medium. Conditioned media of MΦs (RAW-CM) were stored at –80 °C until further analyses.

### 4.8. RNA Isolation and Reverse Transcription

LDSCs and ADSCs at passage 2, as well as RAW 264.7 MΦs cultured in LDSC-CM and ADSC-CM for 48 h, were placed in RNAlater^®^ (Ambion, Life Technologies, Carlsbad, CA, USA) and stored at −80 °C until RNA isolation. The total RNA was isolated from the cells using RNeasy Mini Kit (Qiagen, Venlo, Netherlands) according to the manufacturer’s instructions. During RNA isolation, on-column digestion of residual genomic DNA was performed by DNase I RNase-free set (Qiagen). RNA concentration was determined immediately after isolation using Qubit™ RNA HS Assay Kit (Thermo Scientific, Waltham, MA, USA) on Qubit^®^ fluorimeter (Invitrogen, Thermo Scientific, Waltham, MA, USA), according to the manufacturer’s instructions. Total RNA was reversely transcribed into cDNA using a High-capacity cDNA Reverse Transcription Kit (Applied Biosystems^®^, Foster City, CA, USA), according to the manufacturer’s instructions, with 100 ng per reaction per each sample. Reverse transcription was performed in a PCR thermal cycler SureCycler 8800 (Agilent Technologies, Santa Clara, CA, USA). The protocol conditions were: 10 min at 25 °C, 120 min at 37 °C, 5 min at 85 °C and cooling at 4 °C. The synthesized cDNA was used for relative gene expression analysis.

### 4.9. Real-Time PCR

Quantitative real-time PCR (qPCR) reactions were performed on real time thermal cycler Stratagene Mx3005P (Agilent Technologies, Santa Clara, CA, USA). The qPCR reactions were prepared using a SYBR Fast Universal 2x qPCR Master Mix (Kapa Biosystems, Wilmington, MA, USA), for gene expression analysis in LDSCs and ADSCs, according to the manufacturer’s instruction. For gene expression analysis in RAW 264.7 cells, the qPCR reactions were prepared using Luna^®^ Universal qPCR Master Mix (New England Biolabs, Ipswich, MA, USA). ROX was used as a reference dye. Pre-designed primer sets, consisted of both forward and reverse primers, (QuantiTect primer assay kits) were purchased from Qiagen and were used for the following human genes: *GAPDH* (QT00079247), *CD44* (QT00073549), *POU5F1* (QT00210840), *IL4* (QT00012565), *IL6* (QT00083720), *IL10* (QT00041685), *TNF* (QT00029162) and *ICAM1* (QT00074900) and for the following mouse genes: *Actb* (QT01136772), *Tnf* (QT00104006) and *Il10* (QT00106169). The protocol conditions were: 1) enzyme activation: 3 min (or 1 min) at 95 °C (1 cycle); 2) denaturation: 10 s at 95 °C and annealing/extension (with data acquisition): 30 s at 60 °C (40 cycles). The specific binding of primers was confirmed by melting curve analysis and visualization of specific length products on electrophoresis gel. For LDSCs and ADSCs, the expression level of each target gene was normalized to the glyceraldehyde-3-phosphate dehydrogenase housekeeping gene expression (*GAPDH*) in the same sample, while for RAW 264.7 MΦs the expression level of each target gene was normalized to the β-actin housekeeping gene expression (*Actb*) in the same sample. All analyses were performed by the relative quantification method 2^−∆∆*C*t^. Human (338,112, Qiagen) and Mouse (338,114, Qiagen) XpressRef Universal Total RNA were used as calibrators for qPCR reactions.

### 4.10. Enzyme-Linked Immunosorbent Assays (ELISA)

Secretion of cytokines by RAW 264.7 MΦs cultured for 48 h in LDSC-CM and ADSC-CM, as well as controls, was measured in MΦs’ supernatant by enzyme-linked immunosorbent assays (ELISA). The level of TNF-α, a pro-inflammatory cytokine, was measured by Mouse TNF-alpha Quantikine ELISA Kit (MTA00B, RnD systems, Minneapolis, MN, USA), while level of IL-10, an anti-inflammatory cytokine, was measured by Mouse IL-10 Quantikine ELISA Kit (M1000B, RnD systems, Minneapolis, MN, USA). Both assays were performed according to the manufacturer’s instructions, respectively. Values are expressed as pg of TNF-α or IL-10 per mL.

### 4.11. Nitric Oxide (NO) Measurement

Nitric oxide (NO) was measured in supernatant of RAW 264.7 cells cultured for 48 h in CM of LDSCs and ADSCs and controls, using Griess reagent. Briefly, 100 μL of MΦs’ supernatant was added to an equal volume of Griess reagent and incubated for 10 min at RT. The absorbance was measured at 540 nm. The concentration of NO in the medium was calculated from sodium nitrite (NaNO_2_) standard curve.

### 4.12. L929 Bioassay

The presence of pro-inflammatory cytokines, such as TNF-α, in CM of RAW 264.7 MΦs cultured in LDSC-CM (RAW-CM-LDSC), ADSC-CM (RAW-CM-ADSC), LPS-100 (RAW-CM-L100) and standard medium (RAW-CM) was determined by cytotoxicity evaluation on L929 fibroblasts, the most common cell line used due to high sensitivity to TNF-α [59,60] that results in cell death. In this assay, 2 × 10^4^ cells per well were seeded in 96-well plates in standard medium and after 24 h, different CM were added to the cells. L929 cells cultured in standard medium were used as control. After 24 h cultivation, cells were microscopically analyzed and then MTT test was performed as described above.

### 4.13. In Vitro Wound Healing Assay

To examine the potential in vitro wound healing effect of LDSC-CM and ADSC-CM, we performed a “scratch” test. L929 fibroblasts were seeded in 48-well cell culture plates and incubated in standard cell culture conditions. After reaching the 100% confluence, a wound (“scratch”) was created in cell monolayer. Conditioned media of LDSCs and ADSCs were then added in 50% final dilution. As positive control standard cell culture medium was used (DMEM containing 10% FBS, 2 mM stable glutamine and antibiotic-antimycotic) while in negative control medium serum was omitted. Each sample was tested in four replicates. The “wounds” were incubated with LDSC-CM, ADSC-CM and control media for 3 days in indirect co-culture system in vitro and after that wound closure and cell migration were analyzed on Axio Observer. Z1 inverted light microscope, and morphometric measurements were performed in ZEN 2 (blue edition) software after imaging. Several parameters were monitored and measured: 1) cell migration zone, determined by measuring the area of cell growth and cell migration from the beginning edge of the wound; 2) the wounded area after three days of cultivation in CM and control media; and 3) the extent of wound closure, determined by calculating the ratio between wound surface area three days after cultivation with media and the area of initial wound, before the addition of different media.

### 4.14. Statistical Analysis

All the results were statistically processed and for all samples median as well as mean values were calculated and presented with standard deviation (SD). Results of LDSCs and ADSCs gene expression analyses are presented as scatterplots with median using the templates published by Weissgerber et al. [61]. Statistically significant differences between the samples were analyzed by one-way analysis of variance (ANOVA) and the Mann–Whitney U-test. The value of *p* < 0.05 was considered as significant.

## 5. Conclusions

These are the first data on immunomodulatory and wound healing activity of LDSCs. We showed that both LDSCs and ADSCs are mesenchymal stem cells with similar phenotype and stemness state. Analysis of inflammatory-related genes revealed slightly more pronounced, but not statistically significant, anti-inflammatory character of LDSCs compared to ADSCs. Conditioned media of both LDSCs and ADSCs were shown to be capable of modulating unstimulated RAW 264.7 MΦs’ response in vitro, as evaluated by functional assays on MΦs as well as on gene and protein expression levels, with decreased *Tnf* expression and secretion of TNF-alpha, and increased *Il10* expression. These results suggest that conditioned media of stem cells, with pronounced effect of LDSCs compared to ADSCs, induce anti-inflammatory phenotype of unstimulated RAW 264.7 macrophages. Both LDSC-CM and ADSC-CM showed wound healing activity in vitro comparable with positive control. Based on obtained results we can assume that immunomodulation by lipoma-derived stem cells, through the crosstalk between stem cells and macrophages, may be one of the possible mechanisms involved in lipoma formation.

## Figures and Tables

**Table 1:** NR uptake, NBT and MTT reduction normalized to the cell number obtained by CV test for each sample; results are presented as mean values ± standard deviation (SD).

Type of Conditioned Media (CM)	Ratio between NR Assay and CV Test		Ratio between NBT Test and CV Test		Ratio between MTT Test and CV Test	
**LDSC-CM**	1.25 ± 0.11	*p* = 0.1	1.33 ± 0.18	*p* = 0.06	1.04 ± 0.16	*p* = 0.23
**ADSC-CM**	1.13 ± 0.10		1.12 ± 0.14		0.92 ± 0.14	
